# Adolescence Predictors for Drug Crime Offending: A Follow-up Study of Former Adolescent Psychiatric Inpatients

**DOI:** 10.1007/s10597-020-00708-2

**Published:** 2020-09-10

**Authors:** Mikaela Kontu, Helinä Hakko, Kaisa Riala, Pirkko Riipinen

**Affiliations:** 1 Department of Psychiatry, Research Unit of Clinical Neuroscience, University of Oulu, P.O.Box 5000, 90014 Oulu, Finland; 2 Department of Psychiatry, Oulu University Hospital, Oulu, Finland

**Keywords:** Drug criminality, Substance use, Mental health, Family factors, Young adulthood

## Abstract

Our aim was to examine adolescent predictors (family- and school-related factors, substance use, and psychiatric disorders) for drug crime offending. The initial study population consisted of 508 former adolescent psychiatric inpatients aged between 13 and 17 years. Of them, 60 (12%) had committed a drug crime by young adulthood and they were matched with 120 (24%) non-criminal controls by sex, age and family type. During adolescent hospitalization, study participants were interviewed using valid semi-structured research instruments. Criminal records were obtained from the Finnish Legal Register Centre up to young adulthood. A distant relationship with a father, lying, and thieving, moderate/high nicotine dependence and weekly use of stimulants were shown to be the most prominent predictors for drug crime offending. Our findings encourage the use of modern child- and family-centered approaches in preventing youth involvement in illegal drug use and drug crimes.

## Introduction

Childhood adversity is widely associated with an increased risk of psychiatric disorders in adulthood (Björkenstam et al. 2016), as well as risks of criminal behavior (Baglivio and Epps 2016; Baglivio et al. 2015). It is known that the greater the amount of family-related adversities, the higher the risk is for mental dysfunction (Felitti et al. 2019; Hughes et al. 2017), and criminal behavior in children and adolescents (Baglivio and Epps 2016; Baglivio et al. 2015).

The pathway from childhood behavioral disorders to criminal offending is widely acknowledged in the research literature (Bussing et al. 2010; Fergusson et al. 2005; Pratt et al. 2002). Attention deficit hyperactivity disorder (ADHD), oppositional defiant disorder (ODD), and conduct disorder (CD) are reported to be potential predictors for criminality (Erskine et al. 2016). The effects of these disorders are often mediated by alcohol use and low academic performance during adolescence (Savolainen et al. 2015). CD symptoms are a risk for illicit drug use, both directly and indirectly, through positive attitudes toward illicit drug use (Kolp et al. 2017). Individuals with disruptive behavior disorders (ODD and CD) and ADHD, schizophrenia or nonschizophrenic psychosis (Fazel et al. 2009), and antisocial personality disorder (Black et al. 2010) are shown to be more at risk of engaging in criminal behavior. Most of this excess risk for criminality appears to be mediated by comorbid substance abuse (Fazel et al. 2009). Comorbid substance use and other mental disorders are found to increase the risk for criminal recidivism (Balyakina et al. 2014; Moore et al. 2019). Earlier studies have shown a strong link between substance use and criminal offending (Fazel et al. 2006; Bennet and Holloway 2009; Rezansoff et al. 2013; Moore et al. 2019).

In Finland, the use of major illicit substances among the adult general population has increased over the last decade (EMCDDA 2019a, b, c, d). During the same time, total consumption of alcohol has decreased among people aged 15 and above (Jääskeläinen and Virtanen 2019). In Nordic countries (excluding Iceland), the most commonly used illicit drug is cannabis. In Sweden, Norway and Finland, opioids and amphetamines are the main substances linked to high-risk drug use (EMCDDA 2019a, b, c, d). In Finland, 91% of drug users are poly-drug users, with a preference for opioids and stimulants as primary drugs and cannabis as a secondary drug. There are many social and mental health problems among Finnish drug users, their average level of education is low, and most are unemployed (Kauhanen and Tilhonen 2017). Stenbacka and Stattin (2007) found that adolescents who use illicit drugs have higher levels of criminal activity, compared to those who do not use drugs during adolescence.

Out-of-home placement in adolescence is known to increase the likelihood for psychiatric disorders and criminal convictions in young adulthood (Côté et al. 2018). Family conflicts and parental substance use are related to an increased risk of the offspring experiencing substance use problems in adolescence and young adulthood (Alati et al. 2005; Zhou et al. 2006; Hayatbakhsh et al. 2007; Mezzich et al. 2009). Parental substance misuse is a common social stressor among prisoners with substance use disorders (Grahn et al. 2019). There is also evidence suggesting that criminal offending in adulthood can be prevented with a supportive and non-punitive relationship with parents during adolescence (Johnson et al. 2011), while weak parent–child attachment, and less supervision, are associated with greater involvement in delinquency (Fagan et al. 2011). A direct linkage between bullying and criminal behavior has been shown (Fergusson et al. 2013), but involvement in bullying in any role (bully, victim, or both) is also a notable predictor for criminal behavior and delinquency (Wolke et al. 2013; Lee et al. 2020). Criminality, including drug possession and sales, is more prevalent among young adults who drop out of high school compared those who graduate (Maynard et al. 2015).

This is a register-based follow-up study of former adolescent psychiatric inpatients, who were admitted to psychiatric inpatient care between the ages of 13 and 17. Our focus is on drug crime offending up to young adulthood. The adolescent characteristics of drug crime offenders were compared to those of the age-, sex-, and family-type matched non-criminal controls, identified from the initial sample of adolescents. Adolescent characteristics comprised of family- and school-related adversities, substance use and psychiatric disorders in adolescence. The particular aim was to investigate which of these adolescent characteristics represent the most prominent predictors of drug crime offending up to young adulthood.

## Methods

### Participants

This study is part of a clinical follow-up project, investigating long-term outcomes, and the associations of various psychosocial risk factors, with severe psychiatric and substance use disorders among former adolescent inpatients. The initial study sample consisted of 508 patients (208 male, 300 female), aged between 13 and 17 years, who were consecutively admitted for psychiatric adolescent inpatient care at Oulu University Hospital, during a five-year data collection period from April 2001 to March 2006 (hereafter referred to as index hospitalization). The catchment area of the hospital covers the regions of Oulu and Lapland in Northern Finland, which account for 43% of the total geographical area of the country. Of all adolescents admitted to inpatient care (*n* = 637), subjects who were older than 18 years (*n* = 1), with an organic brain disorders (*n* = 3) or intellectual disability (*n* = 26), or with an inpatient stay too short to complete their interviews (*n* = 22), or who did not give a written, informed consent to participate (*n* = 77), were excluded from the study population. Of all eligible adolescents, 83.7% participated in the study. All study subjects, and their legal guardians, provided written, informed consent for participation in the study. The study protocol is approved by the Ethics Committee of the University Hospital of Oulu (Finland).

### Research Instruments

During index hospitalization, all study participants were interviewed using the Finnish version of Schedule for Affective Disorder and Schizophrenia for School-Age Children Present and Lifetime (K-SADS-PL). The interviews were performed by the treating physician, or a trained medical student under the surveillance of the treating physician. The K-SADS-PL has been shown to be a valid method for defining DSM-IV based psychiatric disorders of adolescents (Kaufman et al. 1997; Ambrosini 2000; Kim et al. 2004). If adolescent information remained uncertain or unreliable, the missing information was obtained from interviews with the parents or guardians. Patients were also interviewed by nurses, using the European Addiction Severity Index (EuropASI) instrument. The EuropASI was used to obtain information on parents’ socio-economic and psychiatric problems (Kokkevi and Hartgers 1995).

### Follow-up Data on Crime

The information on criminality of our study subjects was collected from the Finnish Legal Register Centre. In Finland, a person can be recorded in the criminal register from the age of 15, after having been sentenced to either unconditional or conditional imprisonment, community service, dismissal, a juvenile penalty or a fine in lieu of a juvenile penalty, a fine (supplementary fine) or period of community service or probation, in addition to conditional imprisonment, or a sentence that has been waived on grounds of lack of criminal responsibility (Finnish Legal Register Centre 2019). In our study, the criminal records of the study subjects were available until November 2016.

In Finland it is illegal to import, export, distribute, purchase, manufacture or possess drug-substances that are listed in the drug conventions in the Finnish Narcotic Drugs Act (Finnish Ministry of Justice 2008). This study focuses on drug crime offenders and non-criminal controls and, therefore, the final study sample consisted of 60 drug crime offenders (40 male, 20 female) and 120 non-criminal controls (80 male, 40 female). Of the initial study population, drug crime offenders accounted for 12%, and non-criminal controls accounted for 24%. Drug crime offenders had committed at least one drug crime before November 2016, and the crime type was categorized as either narcotics offence, aggravated narcotics offence, unlawful use of narcotics, or preparation of a narcotics. The mean age for the first drug offence was 20.5 years (SD 2.9 years).

### Adolescent Characteristics

#### Family-Related Factors

Information on family-related factors was obtained from the EuropASI. Family-related factors included family relationships and psychiatric or substance related problems of the family members (mother, father, siblings). Regarding family relationships, the study subjects were asked whether they felt they had a close relationship to each of their family members. Psychiatric or substance related problems of family members were based on the self-report of the study subjects.

#### School-Related Factors

Information on school-related factors was based on the K-SADS-PL. Our focus was on behavioral factors, such as lying, unauthorized absences, starting fights, bullying and thieving. School performance was determined by whether the study subject had ever repeated grades, been moved to an observation class, received special needs education, had an adjusted syllabus or had learning difficulties. Using the EuropASI instrument, the study subjects were asked whether or not they felt they had close friends.

#### Substance Use in Adolescence

The K-SADS-PL was used to gather information on substance use in adolescence. The substances enquired about included alcohol, cannabis, stimulants, sedatives and anxiolytics, opioids, hallucinogens, solvents and inhalants, and other substances. Cocaine and phencyclidine (PCP) were omitted, because none of the study subjects reported ever having used them. In our study, the level of substance use was represented in two ways: use denied or not reported by the study subject and use at least once a week. Information on poly-substance use was also determined.

Information on adolescents’ current smoking habits and the levels of nicotine dependence (ND) were measured using the seven-item modified Fagerström Tolerance Questionnaire (mFTQ). The mFTQ evaluates smoking rate, frequency of inhalation, time between waking up and the first cigarette, level of unwillingness to give up the first cigarette in the morning, difficulty in refraining from smoking in places where it is prohibited, smoking despite medical illness and smoking more in the first two hours of the day than during the rest of the day. The score sum of mFTQ can vary from between 0 and 9, indicating “no ND” (sum score 0–2), “mild ND” (sum score 3–5) and “high ND” (sum score 6–9) (Prokhorov et al. 1996). In our study, the level of nicotine dependence of our study subjects was separated into two groups: no ND and mild/high ND.

#### Psychiatric Disorders

All DSM-IV–based psychiatric diagnoses of the adolescents were assessed using the diagnostic K-SADS-PL interview. In our study, the adolescent psychiatric disorders were categorized into the following major groups: Conduct disorders (DSM-IV: 312.8–312.9, 313.81, 314.00–314.01, 314.9, 299.80), substance-related disorders (DSM-IV: 303.9, 304.0–304.6, 304.8–304.9, 305.0, 305.2–305.7, 305.9), anxiety disorders (DSM-IV: 300.00–300.02, 300.21–300.23, 300.29, 300.3, 308.3, 309.81), affective disorders (DSM-IV: 296.2–296.3, 300.4, 311) and psychotic disorders (DSM-IV: 295, 296.0, 296.4–299.0, 297.1–299.0, 301.13, 301.22). An adolescent can have multiple diagnoses belonging to several psychiatric disorder categories.

The information on the adolescents’ personality disorder (PD) diagnoses (ICD-9: 301, ICD-10: F60.0-0.9) was based on data from the Care Register for Health Care, provided by the Finnish National Institute for Health Welfare (THL), covering up to the end of the year 2016. The instruction from the ICD-10 is that they should not be diagnosed before the age of 16 years, although PDs can be identified in clinical settings in childhood or adolescence. The precise process for PD diagnoses is described in an earlier publication (Kantojärvi et al. 2016).

### Statistical Analyzes

Non-criminal controls were matched with drug crime offenders on age at admission to adolescent psychiatric care ($\pm$ 2 years), gender (exact match), and family type (two parent family and other family types) at admission (exact match). Crude odds ratios (ORs) with 95% confidence intervals (Cl) for drug crime offending were assessed using a binary logistic regression model. Adjusted ORs were calculated using a stepwise binary logistic regression model, and all adolescent characteristics were entered to the model as a potential predictor for drug crime offending. The limit of statistical significance was set at p < 0.05. The statistical software used in our analyses was the IBM SPSS Statistics 25.

## Results

Of all drug crime offenders (*n* = 60), 66.7% were male (*n* = 40) and 33.3% were female (*n* = 20). Table 1 shows family- and school-related factors among drug crime offenders and non-criminal controls. Maternal substance related problems were over two times (OR 2.48; 95% CI 1.02–6.00) more likely among drug crime offenders than non-criminal controls (20.0% vs. 9.2%). A distant relationship with the father was over two times (OR 2.25; 95% CI 1.20–4.24) more common among drug crime offenders than non-criminal controls (58.3% vs. 38.3%).

**Table 1:** Family- and school-related factors among drug crime offenders and non-criminal controls

	Drug crime offenders *n* = 60		Controls *n* = 120		Crude OR	95% CI
	*n*	%	*n*	%		
Family-related factors						
Psychiatric problems						
Maternal	8	13.3	15	12.5	1.08	0.43 − 2.70
Paternal	4	6.7	7	5.8	1.15	0.32 − 4.10
Sibling	4	6.7	6	5.0	1.36	0.37 − 5.00
Substance related problems						
Maternal	12	20.0	11	9.2	2.48*	1.02 − 6.00
Paternal	16	26.7	30	25.0	1.09	0.54 − 2.21
Sibling	3	5.0	9	7.5	0.65	0.17 − 2.49
Distant relationship						
With mother	22	36.7	36	30.0	1.35	0.70 − 2.60
With father	35	58.3	46	38.3	2.25*	1.20 − 4.24
With sibling	19	31.7	34	28.3	1.17	0.60 − 2.30
School-related factors						
Tells lies	41	68.3	43	35.8	3.86***	2.00 − 7.47
Starts fights	40	66.7	44	36.7	3.46***	1.80 − 6.64
Bullies	33	55.9	33	27.5	3.35***	1.74 − 6.42
Thieves	38	63.3	27	22.7	5.89***	2.99 − 11.60
Unauthorized absences	36	60.0	47	39.2	2.33**	1.24 − 4.39
Repeated grade	9	15.0	17	14.2	1.07	0.45 − 2.57
Learning disabilities	23	38.3	40	33.3	1.24	0.65 − 2.37
Observation class	30	50.0	20	16.7	5.00***	2.49 − 10.04
Special needs education	43	71.7	65	54.2	2.14*	1.10 − 4.17
Adjusted syllabus	15	25.0	23	19.2	1.41	0.67 − 2.95
No close friends	12	20.0	26	21.7	0.90	0.42 − 1.95

Significance is *p < .05, **p < .01, ***p < .001

Of school-related factors, drug crime offenders were more likely to tell lies (OR 3.86; 95% CI 2.00–7.47), start fights (OR 3.46; 95% CI 1.80–6.64), bully (OR 3.35; 95% CI 1.75–6.42), thieve (OR 5.89; 95% CI 2.99–11.60) and have unauthorized absences (OR 2.33; 95% CI 1.24–4.39) compared to non-criminal controls. Drug crime offenders were also more likely to be in an observation class (OR 5.00; 95% CI 2.49–10.04) and have special needs education (OR 2.14; 95% CI 1.10–4.17) compared to non-criminal controls.

Table 2 shows substance use in adolescence among drug crime offenders and non-criminal controls. Drug crime offenders were more likely to use alcohol (OR 1.93; 95% CI 1.03–3.62), cannabis (OR 3.92; 95% CI 1.70–9.04), stimulants (OR 14.75; 95% CI 3.18–68.40), sedatives and anxiolytics (OR 8.83; 95% CI 2.76–28.23), opioids (OR 10.41; 95% CI 2.17–49.90) and other substances (OR 13.22; 95% CI 1.55–112.53) at least once a week in adolescence, compared to non-criminal controls. Poly-substance use was more common among drug crime offenders than among non-criminal controls (OR 5.15; 95% CI 1.28–20.70). Drug crime offenders were over five times more likely to have moderate or high level of ND, compared to non-criminal controls (OR 5.48; 95% CI 2.48–12.12).

**Table 2:** Substance use in adolescence among drug crime offenders and non-criminal controls

	Drug crime offenders *n* = 60		Controls *n* = 120		Crude OR	95% CI
	*n*	%	*n*	%		
Substance type						
Alcohol, weekly use	30	50.0	41	34.2	1.93*	1.03–3.62
Nicotine dependence, moderate or high	51	85.0	61	50.8	5.48***	2.48 − 12.12
Drugs, weekly use						
Cannabis	17	28.3	11	9.2	3.92***	1.70 − 9.04
Hallucinogens	2	3.3	1	0.8	4.10	0.37 − 46.19
Stimulants	12	20.0	2	1.7	14.75***	3.18 − 68.40
Sedatives and anxiolytics	14	23.3	4	3.3	8.83***	2.76 − 28.23
Opioids	9	15.0	2	1.7	10.41**	2.17 − 49.90
Other, weekly use						
Solvents and inhalators	6	10.0	4	3.3	3.22	0.87 − 11.89
Others	6	10.0	1	0.8	13.22*	1.55 − 112.53
Poly-substance use, weekly use	7	11.7	3	2.5	5.15*	1.28 − 20.70

Significance is *p < .05, **p < .01, ***p < .001

Table 3 shows adolescent psychiatric disorders and follow-up diagnosis for personality disorders, among drug crime offenders and non-criminal controls. Conduct disorders (OR 6.03; 95% CI 2.85–12.73) and substance-related disorders (OR 4.32; 95% CI 2.22–8.38) were emphasized among drug crime offenders, compared to non-criminal controls. However, the prevalence of psychotic disorders was lower among drug crime offenders compared to non-criminal controls (OR 0.20; 95% CI 0.06–0.69).

**Table 3:** Adolescent psychiatric disorders among drug crime offenders and non-criminal controls

Psychiatric disorders	Drug crime offenders *n* = 60		Controls *n* = 120		Crude OR	95% CI
	*n*	%	*n*	%		
Hospitalization						
Conduct disorders	49	81.7	51	42.5	6.03***	2.85 − 12.73
Substance-related disorders	41	68.3	40	33.3	4.32***	2.22 − 8.38
Anxiety disorders	9	15.0	19	15.8	0.94	0.40 − 2.22
Affective disorders	21	35.0	56	46.7	0.62	0.32 − 1.17
Psychotic disorders	3	5.0	25	20.8	0.20*	0.06 − 0.69
Follow-up						
Personality disorders	10	16.7	10	8.3	2.20	0.86 − 6.62

Comorbidity of adolescent psychiatric disorders is likely; Personality disorders are follow-up diagnoses

Significance is *p < .05, **p < .01, ***p < .001

Table 4 shows the results of stepwise logistic regression analysis, using all adolescent characteristics (family- and school-related factors, substance use in adolescence and adolescent psychiatric disorders) as potential predictors for drug crime offending. Of family- and school related factors, distant relationship with the father (OR 2.60; 95% CI 1.16–5.84), telling lies (OR 2.46; 95% CI 1.10–5.53) and thieving (OR 2.73; 95% CI 1.23–6.06) were associated to drug crime offending. Moderate to high level of ND increased the likelihood for drug crime offending by over threefold (95% CI 1.33–8.48) and the use of stimulants by over eightfold (95% CI 1.54–45.45). Drug crime offenders were less likely to have suffered from affective disorders (OR 0.35; 95% CI 0.15–0.80) or psychotic disorders (OR 0.08; 95% CI 0.01–0.53) during adolescence compared to non-criminal controls.

**Table 4:** The most prominent adolescent predictors for drug crime offending up to young adulthood

	Likelihood for drug crime offending	
	Adjusted OR	95% CI
Family-related factors		
Distant relationship with father	2.60*	1.16 − 5.84
School-related factors		
Tells lies	2.46*	1.10 − 5.53
Thieves	2.73*	1.23 − 6.06
Substance use in adolescence		
Nicotine dependence, moderate/high	3.36*	1.33 − 8.48
Stimulants, weekly use	8.38*	1.54 − 45.45
Adolescent psychiatric disorders		
Affective disorders	0.35*	0.15 − 0.80
Psychotic disorders	0.08**	0.01 − 0.53

The results of a stepwise logistic regression model (Odds Ratio, OR with 95% Confidence interval CI). All variables presented in Tables 1, 2 and 3 are entered to the model as a potential predictor for drug crime offending

Significance is *p < .05, **p < .01, ***p < .001

## Discussion

This register-based follow-up study of former adolescent psychiatric inpatients focused on those who had committed a drug crime by young adulthood. The current study investigated adolescent predictors for drug crime offending, focusing on family- and school-related factors, substance use in adolescence and adolescent psychiatric disorders of the drug crime offenders, compared to their matched non-criminal controls. The results of a final logistic regression model showed that, of all the family-related factors, a distant relationship with the father was emphasized among drug crime offenders. Secondly, drug crime offenders were characterized as being liars and thieves during school-age. Thirdly, drug crime offenders were more likely to have weekly use of stimulants and moderate to high level of ND during adolescence, compared to non-criminal controls. Fourthly, bivariate analyses indicated that drug crime offenders were characterized by conduct and substance-related disorders in adolescence. Affective and psychotic disorders in adolescence were shown to be less common among drug crime offenders compared to controls.

Our study findings revealed that adolescents were more likely to become drug crime offenders if they had a distant relationship with their father, regardless of whether they lived with one or two biological parents, or were raised in out-of-home placements during adolescence. Earlier literature suggests that low quality father-child relationships (Bronte-Tinkew et al. 2006; Hoeve et al. 2009) and paternal absence (Harper and McLanahan 2006) may increase an offspring’s risk of delinquency. However, according to a study on juvenile delinquency by Simmons et al. (2018), having a cruel or uncaring father was shown to increase the risk for adolescent offending more than having an absent father. Considering our finding, we assume that, regardless of the family structure during adolescence, an adolescent can feel emotional distance to their father despite the father’s physical presence. Simmons et al. (2018) also reported that adolescents with a cruel or uncaring father used a greater variety of substances than those with absent fathers. The finding differed from our finding that of the participants with a history of adolescent psychiatric hospitalization, those with a distant paternal relationship were more likely to become drug misusers and drug crime offenders than study participants with a close paternal relationship.

Our study shows that a history of lies telling and thieving at a school-age increased the likelihood for later drug crime offending. Telling lies and thieving are behavioral features that are included in the diagnosis of conduct disorder (Black and Grant 2014). In our study, the prevalence of adolescent conduct disorder among drug crime offenders was twice as high as for non-criminal controls. To justify diagnostic criteria for antisocial personality disorder (ASPD) after the age of 18 years, a patient must have been diagnosed with conduct disorder before the age of 15 years. If the behavioral features of the conduct disorder continue from adolescence into adulthood, the diagnosis becomes ASPD (Black and Grant 2014). Earlier studies have shown that both conduct disorder and ASPD are associated with criminal behavior (Fergusson et al. 2005; Black et al. 2010; Erskine et al. 2016). Therefore, the findings of our study highlight the importance of recognizing those youths for whom ASPD might develop and, consequently, who are at potential risk for involvement in drug criminality. Our novel finding in this study was that telling lies or thieving, per se, associated to drug crime offending. This association appeared regardless of the presence or absence of an adolescent psychiatric disorder.

In our study, moderate to high level of ND and weekly use of stimulants in adolescence were prominent predictors for drug crime offending. There are several studies showing that smoking tobacco in adolescence can increase the likelihood for criminal offending (Aston 2015) and that, in adulthood, smoking tobacco increases the possibility of drug crime offending (Elonheimo et al. 2011). Jurmu et al. (2020) reported that higher levels of ND in adolescence predicted greater levels of drug crime offences committed up to young adulthood. Despite tobacco smoking being illegal for people under 18 years old in Finland (Finnish Ministry of Justice 1976), our findings showed the alarming presence of a significant level of ND already during adolescence. Further, in our study, weekly use of stimulants in adolescence was a substantial predictor for drug crime offending. Earlier studies have reported that stimulants are one of the most misused prescription drugs (Compton and Volkow 2006; Holt and McCarthy 2020), and that non-medical use of stimulants often co-occurs with illicit drug use (Novak et al. 2016). Our study findings suggest that stimulant use in adolescence may also predict drug crime offending, in addition to the use of illicit drugs. However, one limitation of our study was that the information on whether stimulants were prescription drugs or illegal substances, such as amphetamines, was not available.

Among the drug crime offenders in our study, the prevalence of conduct and substance-related disorders in adolescence was twice as high compared to non-criminal controls. However, adolescent affective disorders or psychotic disorders were not emphasized among drug crime offenders. This finding contradicts earlier studies reporting that schizophrenia, nonschizophrenic psychoses and affective disorders were common among people with criminal behavior (Fazel et al. 2008, 2009; Hensel et al. 2020). The proportion diagnosed with personality disorders among drug crime offenders, in our study, was lower than expected. This may be due to under-diagnosis within the health care system (Winsper et al. 2020).

This study has many strengths, including reliable and valid research instruments, such as the K-SADS-PL (Kaufman et al. 1997; Ambrosini 2000), the EuropASI (Kokkevi and Hartgers 1995), and the mFTQ (Prokhorov et al. 1996) for assessing family- and school-related factors, substance use in adolescence, and for defining adolescent psychiatric disorders. In addition, the use of comprehensive data, obtained from the national health and crime registers, made it possible to follow all of the participants up to young adulthood. The nationwide registers have shown to be reliable sources for data in scientific research (Miettunen et al. 2011). The participation rate of the study project was also high (84%).

The current study has also some limitations, however. The information on adolescent substance use, family- (including familial substance use and psychiatric problems) and school-related factors were based on the self-reporting of the adolescents. It should be noted, however, that for family-related factors, adolescents’ self-reports of parental characteristics have been reported as providing valid data for research purposes (Pisinger et al. 2016). Further, the findings of our study cannot be directly generalized to the general adolescent population, because the adolescents participating in our study initially had severe psychiatric disorders requiring inpatient care.

In conclusion, the main findings of our study, suggest that single traits of behavioral disorders in childhood and/or adolescence, together with smoking and illegal substance use, might be proxy markers of later liability for drug crime offending. We believe that child- and family- centered approaches (Niemelä et al. 2019) are an invaluable part of early interventions, in the prevention of illegal drug use and involvement in drug crime offending in adolescence and young adulthood. Family-centered approaches should seek to improve the relationships between distant or absent fathers and their children and families.

## Data Availability

The initial study population consists of adolescent psychiatric inpatients and, therefore, data cannot be released due to ethical and legal reasons.
